# The intrathecal expression and pathogenetic role of Th17 cytokines and CXCR2-binding chemokines in tick-borne encephalitis

**DOI:** 10.1186/s12974-018-1138-0

**Published:** 2018-04-20

**Authors:** Sambor Grygorczuk, Renata Świerzbińska, Maciej Kondrusik, Justyna Dunaj, Piotr Czupryna, Anna Moniuszko, Agnieszka Siemieniako, Sławomir Pancewicz

**Affiliations:** 1 Department of the Infectious Disease and Neuroinfections, Medical University in Białystok, ul. Żurawia 14, 15-540 Białystok, Poland; 2 University Hospital in Białystok, ul. Żurawia 14, 15-540 Białystok, Poland

**Keywords:** Tick-borne encephalitis, Intrathecal inflammation, Neutrophils, Th17, IL-22, CXCL1

## Abstract

**Background:**

Tick-borne encephalitis (TBE) is a clinically variable but potentially severe *Flavivirus* infection, with the outcome strongly dependent on secondary immunopathology. Neutrophils are present in cerebrospinal fluid (CSF) of TBE patients, but their pathogenetic role remains unknown. In animal models, neutrophils contributed both to the *Flavivirus* entry into central nervous system (CNS) and to the control of the encephalitis, which we attempted to evaluate in human TBE.

**Methods:**

We analyzed records of 240 patients with TBE presenting as meningitis (*n* = 110), meningoencephalitis (*n* = 114) or meningoencephalomyelitis (*n* = 16) assessing CSF neutrophil count on admission and at follow-up 2 weeks later, and their associations with other laboratory and clinical parameters. We measured serum and CSF concentrations of Th17-type cytokines (interleukin-17A, IL-17F, IL-22) and chemokines attracting neutrophils (IL-8, CXCL1, CXCL2) in patients with TBE (*n* = 36 for IL-8, *n* = 15 for other), with non-TBE aseptic meningitis (*n* = 6) and in non-meningitis controls (*n* = 7), using commercial ELISA assays. The results were analyzed with non-parametric tests with *p* < 0.05 considered as significant.

**Results:**

On admission, neutrophils were universally present in CSF constituting 25% (median) of total pleocytosis, but on follow-up, they were absent in most of patients (58%) and scarce (< 10%) in 36%. CSF neutrophil count did not correlate with lymphocyte count and blood-brain barrier integrity, did not differ between meningitis and meningoencephalitis, but was higher in meningoencephalomyelitis patients. Prolonged presence of neutrophils in follow-up CSF was associated with encephalitis and neurologic sequelae. All the studied cytokines were expressed intrathecally, with IL-8 having the highest CSF concentration index. Additionally, IL-17A concentration was significantly increased in serum. IL-17F and CXCL1 CSF concentrations correlated with neutrophil count and CXCL1 concentration was higher in patients with encephalitis.

**Conclusions:**

The neutrophil CNS infiltrate does not correlate directly with TBE severity, but is associated with clinical features like myelitis, possibly being involved in its pathogenesis. Th17 cytokine response is present in TBE, especially intrathecally, and contributes to the CNS neutrophilic inflammation. IL-8 and CXCL1 may be chemokines directly responsible for the neutrophil migration.

**Electronic supplementary material:**

The online version of this article (10.1186/s12974-018-1138-0) contains supplementary material, which is available to authorized users.

## Background

Tick-borne encephalitis (TBE) is caused by a *Flavivirus* species (TBE virus, tick-borne encephalitis virus (TBEV)) transmitted by Ixodes ticks. The European TBEV subtype frequently causes a mild flu-like disease, but in some cases, it is able to penetrate the blood/brain barrier (BBB) causing the second, neurologic phase of the disease, which may take a severe course and leave permanent neurologic sequelae [1–5**]**. Both the animal models and the results of human studies point to the involvement of the host immune/inflammatory response in the pathogenesis. TBEV entry into the central nervous system (CNS) probably occurs at least partially via transcellular pathway [6], but animal models of *Flavivirus* encephalitis suggest it may be facilitated initially by the disruption of BBB by the excessive systemic inflammation [7, 8] and subsequently by the virus-infected leukocytes migrating to the intrathecal inflammatory focus [9]. The further CNS tissue damage results from a combination of TBEV cytopathic effect on neurons [10] and of a vivid intrathecal inflammatory response, involving primarily Th1-subtype CD4+ and cytotoxic CD8+ lymphocytes [11–15]. Interestingly, mouse strains more sensitive to TBEV present with a higher expression of the pro-inflammatory cytokines and chemokines during encephalitis [16].

The cerebrospinal fluid (CSF) of TBE patients is characterized by the increased albumin concentration and albumin CSF/serum index persisting during the convalescent period, indicating protracted BBB disruption, elevated IgG index pointing to the intensive intrathecal humoral response [17], and a tendency for a relatively low initial pleocytosis with a high neutrophil fraction [4, 18]. The neutrophils may constitute a majority of CSF leukocyte population at the initial diagnostic examinations, analogously to the infections with related neurotropic *Flavivirus* species, West Nile virus (WNV) and Japanese encephalitis virus (JEV) [19, 20]. This is typically accompanied by a moderate peripheral neutrophilia, a likely source of cell population penetrating into CSF. However, the factors driving this neutrophilic response and its role in the pathogenesis are not well characterized.

Neutrophils are involved in the early unspecific response and elimination of pathogens, which is typically accompanied by a significant local tissue damage [21, 22]. Through the secretion of cytokines including interleukin-12 (IL-12) and chemokines acting preferentially on Th1 (CXCL9, CXCL10, CXCL11) and Th17-lymphocytes (CCL2, CCL20), they participate in the initiation and shaping of the later stages of the response, favoring the development of Th1 and/or Th17-type milieu [21–24]. Data from animal studies show that neutrophils are involved mainly during the onset of viral meningitis/encephalitis, after which they are replaced by mononuclear cells [25–28]. In WNV infection in mice, they are upregulated both in periphery and in CNS at the onset of encephalitis analogously to human TBE [28]. In this model, infected neutrophils play an important pathogenic role as an early reservoir and a vehicle of virus entry into CNS and were found to co-localize with WNV in meninges, thus contributing prominently to the onset and severity of encephalitis [9, 27]. Later, during the established encephalitis, they seem to contribute to its control and, accordingly, their depletion is beneficial when occurring directly before, but harmful after an exposition to the virus [27]. The balance of the protective and harmful effects of the neutrophil infiltration in TBE is unknown and the existing data on other human *Flavivirus* infections are few and conflicting. For example, the activation of neutrophils in contact with JEV and an effective degradation of viral proteins and RNA has been observed in vitro suggesting a protective role [20], but intrathecal expression of a chemokine for neutrophils, IL-8, is associated with a poor prognosis in Japanese encephalitis (JE) in vivo [29]. Intrathecal neutrophils should influence the development of the next stages of the local inflammatory/immune response, which is consistent with the prevailing Th1-type response observed in TBE [13, 14, 30], and hints at the possibility of the coexistence of Th17-mediated response as well.

The Th17-type specific response, essential for the development of a sustained neutrophil-dominated inflammation, is driven by a subset of immune cells (including γδ lymphocytes, a differentiated Th17 subpopulation of classical CD3+CD4+ lymphocytes, and a specialized fraction of NK cells) able to secrete a set of cytokines including interleukin-17A (IL-17A), IL-17F, and IL-22 [23, 31–36]. Th17-type cytokine response may be boosted in an autocrine/paracrine manner by IL-17 acting on CD4+ lymphocytes [34]. Having no receptors directly on neutrophils, IL-17 and IL-22 promote a neutrophil-dominated inflammation indirectly via mediators released from tissue cells like hepatocytes, epithelial, and endothelial cells [9, 23, 37]. Especially, they upregulate the expression of the subset of chemotactic cytokines (chemokines) specific for neutrophils, including IL-8 (CXCL8), CXCL1, CXCL2, and CXCL5, signaling through receptors CXCR1 (binding mainly IL-8) and CXCR2 (binding IL-8, CXCL1, CXCL2, and CXCL5) [23, 36–40]. The neutrophils support the activation and migration of Th17 cells in a positive feedback loop, with the resultant response having a strong pro-inflammatory and self-sustaining character [23]. These characteristics make Th17-dependent inflammation deeply involved in the pathogenesis of autoimmunity, which was especially well documented in autoimmune arthritis [34, 41, 42].

Although originally linked to the response against extracellular pathogens in skin and mucous membranes, Th17 cells may contribute to the inflammation and immunopathology within CNS. IL-17 and IL-22 act on BBB endothelial cells, increasing secretion of IL-6, CCL2, CXCL1, CXCL2, and IL-8; increasing BBB permeability; and facilitating transmigration of CD4+ cells [9, 43]. In that way, they may participate in the onset of encephalitis promoting BBB disruption and CNS inflammation. Moreover, human Th17 lymphocytes preferentially transmigrate across BBB and may have a cytolytic effect on neurons through the expression of granzyme B, directly contributing to the CNS damage [43]. The role of Th17 response in human CNS pathology was confirmed in multiple sclerosis [43, 44], and increased concentrations of CXCL1, IL-8, and granulocyte colony-stimulating factor (G-CSF) were detected in CSF of patients with acute disseminated encephalomyelitis [45]. In TBE and other *Flavivirus* neuroinfections, the involvement of Th17 lymphocytes and related cytokines might explain both the presence of the substantial neutrophil infiltrate and the significant immunopathology. The study by Wang et al. on WNV-infected mice confirmed the intrathecal expression and pathogenetic role of IL-22 and CXCL1 [9]. Pietikäinen et al. detected highly increased concentrations of CXCL1, IL-8, IL-17, and G-CSF in CSF of a group of TBE patients [46]. IL-8 was also detected in TBEV-infected astrocytes and in the serum of TBE patients, but its chemotactic gradient towards CSF in vivo has not been directly studied [14, 47].

We have attempted to gather more data on the role of neutrophils in the pathogenesis of TBE in humans and on the expression of the cytokines which might contribute to their recruitment into CNS. We have analyzed retrospectively the correlations between the CSF neutrophil count and other laboratory and clinical parameters, including the disease manifestation and severity, in TBE patients hospitalized in six consecutive years in a reference center. We have also analyzed concentrations of chemokines for neutrophils IL-8, CXCL1, and CXCL2, as well as Th17 cytokines IL-17A, IL-17F, and IL-22 in CSF and serum in prospectively recruited TBE patients, to assess their peripheral and intrathecal expression, concentration gradients, and relation to the CSF inflammatory parameters.

## Methods

### Patients

We have studied TBE patients hospitalized in the Department of the Infectious Diseases and Neuroinfections of the Medical University of Białystok. The initial diagnosis was based on the history of a recent tick bite or exposition to ticks in TBE endemic areas, clinical symptoms, and CSF pleocytosis and confirmed with the detection of the specific anti-TBEV IgM antibodies in serum and/or CSF on admission or by the time of discharge from the hospital. Most of the patients underwent two lumbar punctures: on admission to hospital and before the discharge 10–16 days later. Patients with residual clinical symptoms or protracted inflammatory changes in CSF had additional control lumbar punctures later in the convalescent period, typically 4–8 weeks after admission.

The data from the records of patients hospitalized between 2009 and 2014 were analyzed in the retrospective part of the study. Only patients with CSF pleocytosis ≥ 15 cells/μl on admission were included, which excluded patients with peripheral TBEV infection without CNS involvement. Patients in whom the microscopic CSF evaluation on admission was not performed, because of low CSF cytosis or for technical reasons, in whom the original CSF examination was done outside of our department or with a delay of ≥ 3 days after admission, were not included either. This left 240 patients (150 women, 90 men, aged from 18 to 81 years old, mean age 47 years) in whom we have analyzed associations of the peripheral neutrophilia and CSF neutrophil count with the clinical course, manifestation, severity, outcome, presence of an altered mental status, and paresis, as well as with basic laboratory parameters: C-reactive protein (CRP) concentration, blood leukocytosis and lymphocytosis, CSF pleocytosis, lymphocytosis, total protein, and albumin concentration.

TBE with a classical biphasic course was defined as a disease with a flu-like peripheral phase with transient improvement followed by a neurologic phase, as opposed to a monophasic onset of a neurologic disease. Meningoencephalitis (ME) was diagnosed based on the altered mental status and/or focal neurologic deficits, meningoencephalomyelitis (MEM) based on sensory or motor symptoms from the spinal nerves, and patients not fulfilling the ME or MEM criteria were diagnosed with meningitis (M). The disease severity was scored in a pre-defined scale from 1 to 6, in which 1 corresponded to an evidently uncomplicated meningitis; 2 very mild or dubious CNS involvement with no altered mental status nor paresis (limited, transient neurologic symptoms, e.g., pyramidal signs, paresthesia, tremor); 3 mild encephalitis with lethargy, drowsiness, emotional lability, monofocal paresis, gait disorders, but patients remaining in a logical contact and able to walk; 4 moderately severe encephalitis with disorientation, abnormal behavior, multifocal and/or severe neurologic symptoms, and generalized seizures; 5 severe encephalitis with loss of consciousness; 6 coma or death. The mental status was scored from 0 (normal) through 1 (mild abnormalities like apathy, impaired concentration), 2 (moderate abnormalities––disorientation, confusion, agitation) to 3 (lack of consciousness). The outcome in surviving patients (as noted on discharge from hospital or during control visits) was scored from 0 to 3 (0—no signs or symptoms on discharge or follow-up, 1—only subjective complaints, 2—objective neurologic deficits, and 3—disabling sequelae). The division of the patient cohort according to the above criteria is shown in Table 1.

**Table 1:**
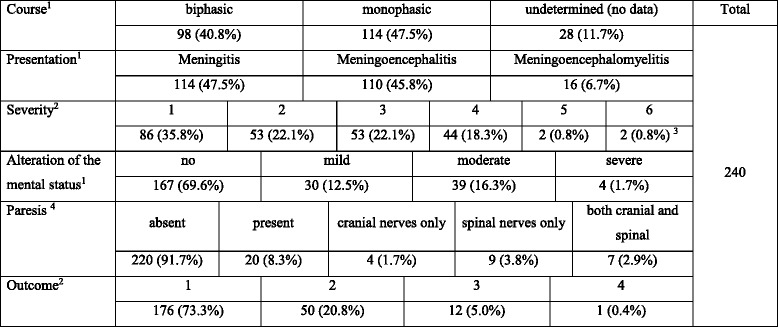
The clinical characteristics of the retrospective study cohort

^1^groups defined as described in Material and methods;

^2^scored in a pre-defined scale, as described in Material and methods;

^3^including one fatal case;

^4^not summing up to 100%

Six patients were diagnosed by a treating physician with a probable or confirmed neuroborreliosis coexisting with TBE, based on a positive serology and intrathecal synthesis of the specific antibodies. The patients came from the area endemic for Lyme borreliosis and could be seropositive against *Borrelia burgdorferi* after previous symptomatic or asymptomatic infection, which makes the diagnosis of an active disease challenging. In the group co-infected with TBEV, it is especially problematic as the assessment of intrathecal synthesis of the specific antibodies against *Borrelia burgdorferi* is complicated by the acute BBB disruption. Neither the clinical presentation nor laboratory parameters differed systematically in these patients from the rest of the group, which led us not to exclude them from the study.

In the prospective pilot study, IL-8 serum concentration was measured on admission to hospital in 36 patients with TBE hospitalized in 2014 and 2015, to confirm previous findings on IL-8 expression in TBE and study its specificity for TBE versus other viral meningitides [47]. Of these, 13 patients had uncomplicated meningitis, 21 presented with ME, and 2 presented with brachial paresis were diagnosed with MEM. CSF concentration was measured in 5 patients: 3 in M and 2 in ME group.

The main prospective study group consisted of 15 patients hospitalized in 2015 and 2016, 11 with M and 4 with ME, of whom 3 had mild presentation with mainly cerebellar symptoms and 1 moderately severe presentation with an altered mental status. Eight patients had biphasic and 7 monophasic disease. The data of this group are presented in Table 2.

**Table 2:** The basic clinical and laboratory data of the TBE patients included in the prospective study

	Sex	Age	Presentation	Altered consciousness	Neurologic symptoms	CRP^1^	Blood morphological parameters			CSF parameters					*Q*_alb_
							Leukocytosis^2^	Neutrophils^2^	Lymphocytes^2^	Pleocytosis^2^	Neutrophils^2^	Lymphocytes^2^	Protein^3^	Albumin^3^	
1	f	69	M	No	No	0.2	9260	6550	1790	23	4	18	41.1	33.1	0.07
2	m	27	M	No	No	5.2	3150	1090	1420	235	45	139	67.7	51.2	0.12
3	m	61	M	No	No	12.2	12,100	10,060	1620	48	0	46	54.6	38.5	NA
4	f	53	M	No	No	0.4	5650	7170	2040	120	0	116	59	58.3	0.14
5	f	43	EM	No	Ataxia	1.3	7120	4380	1550	144	68	62	47	33	0.08
6	f	68	EM	Lethargy	Ataxia	2.6	5160	4220	610	241	174	55	47.3	36.5	0.09
7	m	24	M	No	No	24.8	17,200	14,810	1080	142	48	89	116.4	87.4	0.18
8	f	36	EM	No	Tremor	54.2	14,000	NA	NA	236	35	177	130	99.9	0.24
9	f	70	EM	No	Cerebellar syndrome, tremor	27.6	5860	4350	980	171	84	87	101.7	85.7	0.23
10	m	20	M	No	No	35.3	13,620	10,200	1630	71	28	30	25.3	16.5	0.04
11	f	41	M	No	No	29.1	7950	5880	1320	103	5	93	65	47.8	0.11
12	m	42	M	No	No	0.2	6680	4430	1120	44	3	35	31	20.2	0.06
13	f	27	M	No	No	33.3	10,270	8430	1220	424	89	331	86.7	56.8	0.13
14	m	26	M	No	No	56.2	11,240	9170	1050	260	88	146	65	49.3	0.11
15	m	30	M	No	No	10.8	13,220	11,260	1010	194	62	107	77.3	58.2	0.12

*f* female, *m* male, *M* meningitis, *ME* meningoencephalitis, *CRP* C-reactive protein, *CSF* cerebrospinal fluid in the active neurologic phase of the infection, *Q*_*alb*_ CSF/serum albumin quotient, *NA* non-available

^1^In mg/l

^2^In cells/μl

^3^In mg/100 ml

As the control samples, we used sera from 7 healthy blood donors and CSF from 7 patients in whom neuroinfection was ruled out by means of a lumbar puncture. Additionally, concentrations of IL-8, CXCL2, IL-17A, and IL-17F were studied in paired serum and CSF samples obtained on admission from 6 patients with non-TBE lymphocytic meningitis hospitalized during Echovirus 30 meningitis outbreak.

The subjects included in the prospective study gave informed consent for the entry, and the study was accepted by the Bioethics Committee of the Medical University of Białystok (ref. no of the approval decisions R-I-002/66/2014 and R-I-002/225/2015).

### Sample collection

The blood and CSF samples for the prospective study were obtained simultaneously, together with the materials for clinically indicated laboratory examinations: examination I on admission to hospital, examination II before discharge, and examination III during the later control visits in the convalescent period.

In the IL-8 group, only 5 patients (the same in whom the CSF was studied) were studied at the examination II time point. In the main study group, the examination I samples were available in all TBE patients and examination II samples in 13 patients (only IL-22 and CXCL1 were measured in one of them at examination II time point because of the limited CSF volume). In 5 patients, the later convalescent period (examination III) paired serum and CSF samples were additionally studied for the concentration of IL-22 and CXCL1, to assess the long-term dynamics of the pro-neutrophilic cytokine response. Two patients (patients 1 and 2 in Table 2) were initially hospitalized and had the first lumbar puncture performed during, what eventually appeared to be, a peripheral phase of TBEV infection and were subsequently re-investigated during neurologic phase (14 days later). Patients 3 and 4 underwent the first puncture at the onset of the neurologic phase when they had minor CSF abnormalities (pleocytosis of 17 and 27 cells/μl, respectively), but subsequently deteriorated clinically and had a lumbar puncture repeated within a week, revealing much more pronounced CSF inflammatory changes, as listed in Table 2. In these 4 patients, the blood and CSF obtained during the maximum CSF inflammation were considered the examination I samples, while the pre-neurologic phase samples were included in the study, but analyzed separately.

The venous blood samples were collected to EDTA-coated tubes and centrifuged for serum collection within half an hour. The CSF samples were collected into sterile tubes and stored for no longer than 24 h in 5–6 °C. Subsequently, serum and CSF samples were, without additional processing, frozen and stored in − 80 °C. All the samples were thawed and analyzed for the cytokine concentrations simultaneously, one run of each measurement performed per sample.

### Laboratory techniques

Peripheral leukocytosis, CRP concentration, and serum albumin concentration as well as basic CSF parameters (total pleocytosis, neutrophil and lymphocyte count, total protein and albumin concentration) were studied with standard laboratory techniques. Anti-TBEV IgM antibodies in serum and CSF were detected with Enzygnost Anti-TBE/FSME IgM kit from Siemens (Munich, Germany), following the standard procedure.

Concentrations of cytokines were measured with commercial ELISA kits, strictly following the manufacturer’s instructions: CXCL1 and IL-8 with a Quantikine® ELISA from R&D Systems (Minneapolis, USA), IL-22 with IDELISA™ from ID Labs Inc. (London, Canada), IL-17A, IL-17F, and CXCL2 from Shanghai Sunred Biological Technology Co. (China). The sensitivity of the assays according to the manufacturers’ data was 10 pg/ml for CXCL1, 5 pg/ml for CXCL2, 3.5 pg/ml for IL-8, 0.5 pg/ml for IL-17A, 0.5 pg/ml for IL-17F, and 10 pg/ml for IL-22. Any read-out falling below that level was considered 0 in the analysis.

CSF albumin quotient (*Q*_alb_) was calculated as albumin_CSF_/albumin_serum_. Because of the lacking data on serum albumin concentrations at the day of the first lumbar puncture, it could be calculated in only 36 patients (15%) of the retrospective cohort and in 14 out of 15 in the prospective cohort. The indexes of cytokine concentrations in CSF were calculated as *I* = (cytokine_CSF_/albumin_CSF_)/(cytokine_serum_/albumin_serum)_ in 14 prospective cohort patients in whom the serum albumin concentration was known.

### Statistical analysis

The data were analyzed with Statistica 10 software with the use of non-parametric tests and *p* < 0.05 was considered statistically significant. In both parts of the study, Mann-Whitney *U* test was used for the two-group comparisons (patients with and without paresis or altered consciousness, cytokine concentrations in study, and control groups), Kruskal-Wallis non-parametric ANOVA for comparisons of multiple groups (patient groups with different severity, consciousness, and outcome scores), *R* Spearman test for the analysis of correlations, and chi-square test for the analysis of the distribution of the scored variables. The intra-group comparisons (difference between serum and CSF concentrations and between examination I and II values) were performed with Wilcoxon pair test.

In the retrospective study, patients with the most severe presentation or sequelae were too few to be included in the formal statistical analysis. Also, not all data were available for all the patients (especially on follow-up), decreasing the case numbers in particular analyses.

## Results

### The retrospective study

#### The time since onset

The patients were hospitalized at different times after the disease onset, which could have influenced the peripheral and CSF inflammatory parameters, possibly confounding the results. To assess this, we have noted the approximate time intervals from (1) tick bite; (2) onset of symptoms; and (3) onset of fever, to the lumbar puncture, whenever there were sufficient data in the medical records and checked for the association with the admission laboratory parameters. There was no correlation between any of the analyzed intervals and any of the studied CSF parameters, blood leukocyte count, and CRP concentration, meaning they were not critically dependent on the duration of the disease. There was only a weak (*R* = − 0.28) but significant (*p* < 0.005) decrease of the peripheral blood neutrophil count with the time since the onset of fever (Additional file 1).

#### Peripheral blood neutrophil count

**Fig. 1:**
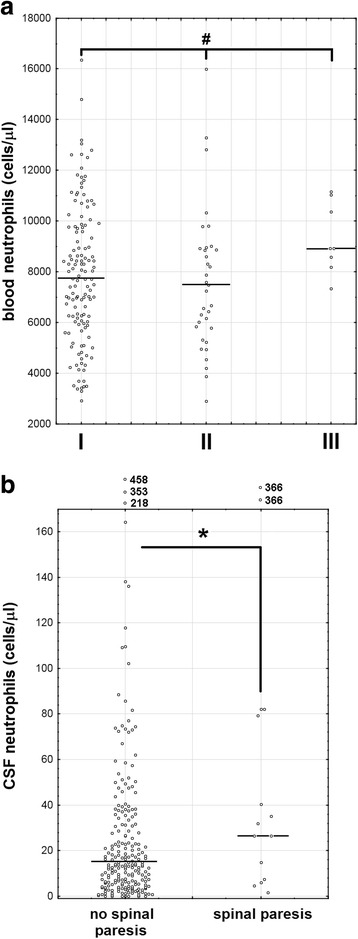
The initial blood and cerebrospinal fluid neutrophil counts in tick-borne encephalitis, dependent on clinical features. **a** The peripheral blood neutrophils (cells/μl) in patients stratified by outcome: no sequelae (I, *n* = 133), only subjective complaints (II, *n* = 34), objective neurologic deficits (III, *n* = 8). ^#^A trend with a statistical significance at *p* = 0.0504 with non-parametric ANOVA. **b** The cerebrospinal fluid neutrophils (cells/μl) in patients with (*n* = 15) and without (*n* = 225) spinal paresis. *A significant difference between the groups, *p* < 0.05. The data from individual patients are shown with circles, median values with horizontal lines. Five extremely high values in **b** are shown out of scale above the plot

TBE patients presented with mild leukocytosis and elevated neutrophil count as well as mildly increased CRP concentration, independent of the clinical presentation (Table 3). Leukocytosis and neutrophilia were significantly (by about 1000/μl) higher in women than in men (not shown). The full blood differential with neutrophil count was available in 176 patients. The patients with the most severe presentation tended to have higher neutrophilia, but the trend did not reach the level of the statistical significance. However, of 5 patients with neutrophilia exceeding 13,000/μl, all had either altered consciousness or evident neurologic deficits, resulting in the highest recorded neutrophil count of 16,360/μl in ME versus 12,820/μ in M group (Table 3). There was also a tendency for higher neutrophilia in patients who eventually developed long-lasting neurologic deficits (*p* = 0.054, Fig. 1a).

**Table 3:** The median, quartile (in parentheses), and extreme [in brackets] values of peripheral blood parameters assessed in the retrospectively analyzed group of 240 patients with tick-borne encephalitis virus infection, stratified by clinical presentation

	Leukocytosis (cells/μl)^a^	Neutrophil count (cells/μl)^b^	Lymphocyte count (cells/μl)^c^	CRP concentration (mg/l)^d^
Total (*n* = 240)	10,220 (8415–12,170) [3300–20,700]	7729 (6023–9651) [2902–16,361]	1464 (1188–1788) [527–3475]	8.7 (3.0–16.5) [0.0–96.0]
M (*n* = 114)	10,040 (8520–12,300) [3300–20,700]	7597 (6204–9512) [2913–12,820]	1503 (1218–1963) [669–3475]	8.1 (2.35–15.0) [0.0–96.0]
ME (*n* = 110)	10,400 (8105–11,850) [3600–19,350]	7693 (5942–9620) [2902–15,983]	1360 (1136–1793) [656–3142]	10.1 (3.6–18.0) [0.0–51.7]
MEM (*n* = 16)	10,385 (9330–12,083) [5660–17,350]	8185 (7330–10,353) [3702–16,361]	1233 (828–1683) [527–2770]	8.6 (2.05–14.0) [0.0–61.5]

*CRP* C-reactive protein, *M* meningitis, *ME* meningoencephalitis, *MEM* meningoencephalomyelitis

^a^Reference values 4000–10,000

^b^Reference values 1600–7200

^c^Reference values 800–4700

^d^Reference value < 5 mg/l

#### CSF neutrophils on admission

The CSF parameters in the study cohort are shown in Table 4. Neutrophils were detectable in CSF of 97% of patients, their fraction varying from 1 to 93% of total pleocytosis (median 25% for the whole group, 25% in M, 24% in EM, and 39% in MEM). The CSF inflammatory parameters did not differ between the M and ME groups, while the MEM group stood out with a significantly (*p* < 0.05) higher neutrophil fraction, neutrophil count, total protein, and albumin concentration. The CSF neutrophil count did not differ between patients with monophasic and biphasic course of the disease and did not correlate with the score of the disease severity, consciousness level, or outcome (not shown). However, it was significantly higher in patients with paresis (*p* < 0.05) and especially in patients with a spinal paresis in comparison with the rest of the cohort (*p* < 0.03) (Fig. 1b).

**Table 4:** The median, quartile (in parentheses), and extreme [in brackets] values of the cerebrospinal fluid parameters in the retrospectively analyzed group of 240 patients with tick-borne encephalitis virus infection dependent on the clinical presentation

	Examination I					Examination II^1^			
	Pleocytosis (cells/μl)	Neutrophil count (cells/μl)	Protein (mg/dl)	Albumin (mg/dl)	*Q*_alb_	Pleocytosis (cells/μl)	Neutrophil count (cells/μl)	Protein (mg/dl)	Albumin (mg/dl)
Total (*n* = 240)	79 (49–138) [15–837]	14 (6–35) [0–458]	61 (49–77) [21–168]	43 (35–53) [15–99]	0.10 (0.08–0.13) [0.06–0.21]	43 (30–64) [9–174]	0 (0–1) [0–89]	58 (45–76) [19–209]	40 (30–54) [12–169]
M (*n* = 114)	80 (51–146) [16–815]	14 (6–34) [0–353]	59 (46–70) [21–121]	42 (33–50) [15–97]	0.09 (0.08–0.11) [0.06–0.17]	43 (24–57) [10–174]	0 (0–1) [0–89]	57 (43–73) [19–205]	38 (29–50) [12–165]
ME (*n* = 110)	77 (44–120) [15–837]	14 (5–27) [0–458]	64 (49–80) [27–168]	41 (35–54) [17–99]	0.10 (0.09–0.13) [0.07–0.21]	47^b^ (34–71) [11–140]	0.38 (0–1) [0–23]	58 (47–80) [20–209]	40 (31–57) [12–169]
MEM (*n* = 16)	98 (52–256) [34–475]	34^a^ (13–82) [3–366]	74^b^ (63–87) [50–108]	53^b^ (43–66) [33–71]	0.20	22^c^ (17–35) [9–59]	0.25 (0–2) [0–2]	81^a^ (68–126) [47–203]	51^b^ (41–87) [31–135]

*M* meningitis, *ME* meningoencephalitis, *MEM* meningoencephalomyelitis, *Q*_*alb*_ CSF/serum albumin quotient (data available in a subgroup of 36 patients: 18 with M, 17 with ME, and one with MEM)

^1^Full data available in 166 patients of the cohort

^a^Significantly higher than in M and ME groups

^b^Significantly higher than in M group

^c^Significantly lower than in M and ME groups

**Fig. 2:**
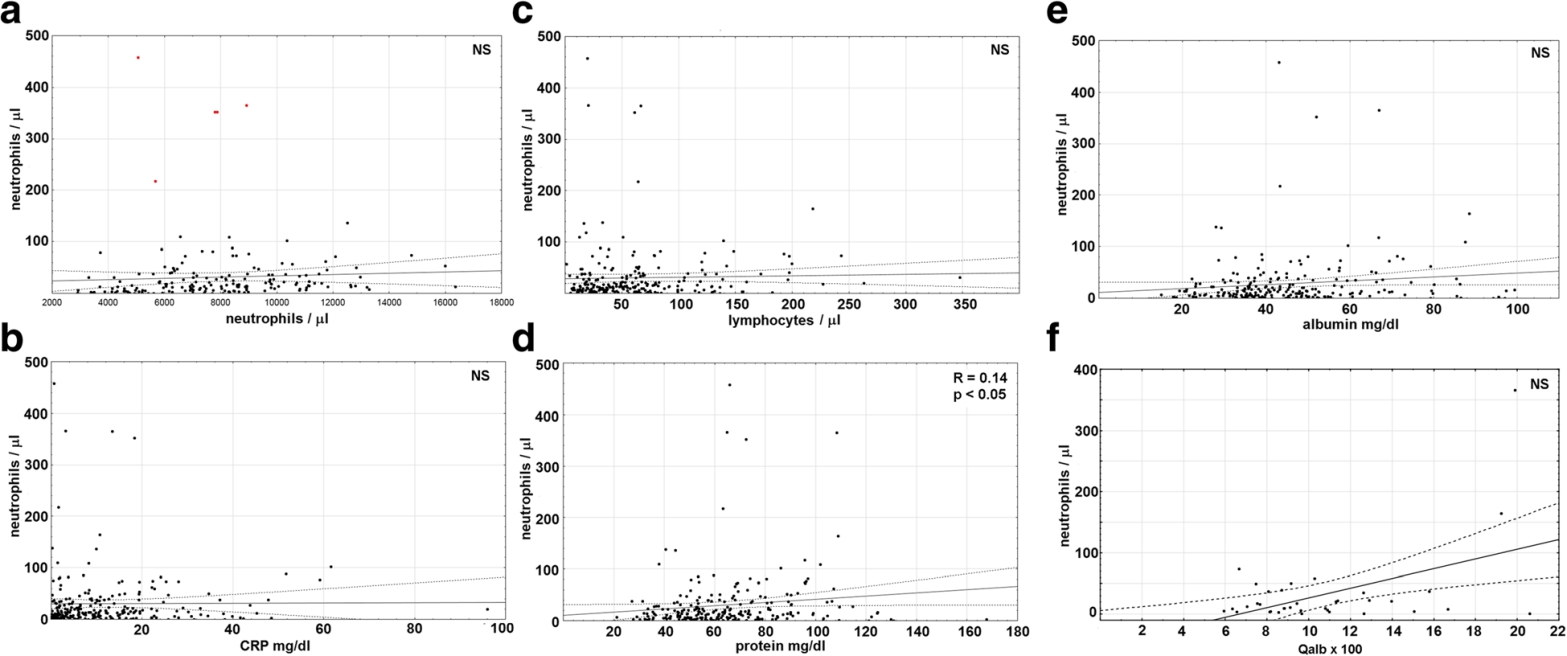
The correlation of the cerebrospinal fluid neutrophil count with the peripheral and intrathecal inflammatory parameters. The laboratory parameters on admission to hospital in a group of 240 patients with TBE. The neutrophil count expressed in cells/μl is presented on the vertical axis in all the panels plotted against other parameters presented on horizontal axes. **a** Peripheral blood neutrophilia (in cells/μl). **b** Serum concentration of C-reactive protein (CRP, expressed in mg/l). **c**–**e** Cerebrospinal fluid (CSF) inflammatory parameters: **c** lymphocyte count (cells/μl), **d** total protein concentration (mg/dl), and **e** albumin concentration (mg/dl). **f** The CSF/serum albumin quotient (*Q*_alb_) calculated as described in the “Methods” section in a subgroup of 36 patients. The data from individual patients are shown with points, the linear fit with a continuous line and the 95% confidence interval with dashed lines. For significant correlations, the strength *R* and significance *p* are presented in the upper right corner of a panel. NS non-significant. In **a**, 5 extreme cases with a very high CSF neutrophil counts exceeding 200/μl, exclusion of which revealed a significant correlation, are highlighted with red

The CSF neutrophil counts presented with a non-significant trend for a positive correlation with blood neutrophilia, which reached a level of a statistical significance only when 5 patients with extremely high counts (> 200/μl) were excluded (*R* = 0.32, *p* < 0.001) (Fig. 2a). It did not correlate with C-reactive protein concentration in serum (Fig. 2b), with CSF lymphocyte count, CSF albumin concentration, and correlated only weakly with CSF total protein concentration (*R* = 0.14, *p* < 0.05) (Fig. 2c–e). In a subgroup of patients in whom *Q*_alb_ could have been calculated, it apparently tended to correlate non-significantly with the CSF neutrophil count, but the trend was totally dependent on a single extreme case (Fig. 2f).

#### CSF neutrophils on follow-up

The examination II pleocytosis was significantly lower in MEM than in M and ME groups (*p* < 0.05), while the protein and albumin concentrations remained higher in MEM (*p* < 0.05, Table 4). The CSF neutrophil counts tended to normalize faster than the other parameters. The CSF infiltrate in 58% of patients was purely mononuclear; in 36%, the neutrophils were detectable, but scarce (from typically 1–2 up to 10%) and only in the remaining 6% there remained a neutrophil infiltrate exceeding 10% of total pleocytosis. These residual neutrophils were present less frequently in M in comparison with ME and with combined ME and MEM groups (*p* < 0.01) (Table 5). There was no correlation between the admission and follow-up neutrophil counts.

**Table 5:** The presence of the residual neutrophils in the cerebrospinal fluid of 172 TBE patients in the convalescent period after 10–16 days of hospital treatment associates with the clinical presentation

	TBE total *n* = 172	TBE presentation			
		M *n* = 86	ME *n* = 78	MEM *n* = 8	ME + MEM *n* = 86
Neutrophils absent (patient number and percentage)	99 58%	58^a, b^ 68%	37^a^ 47%	4 50%	41^b^ 48%
Neutrophils present (patient number and percentage)	73 42%	28^a, b^ 32%	41^a^ 53%	4 50%	45^b^ 52%

Shown are the numbers and the percentages of patients with and without cerebrospinal fluid neutrophils

*M* meningitis, *ME* meningoencephalitis, *MEM* meningoencephalomyelitis

^a^Significant difference between M and ME groups (*p* < 0.01) in a chi-square test

^b^Significant difference between M and combined ME/MEM group (*p* < 0.01) in a chi-square test

**Fig. 3:**
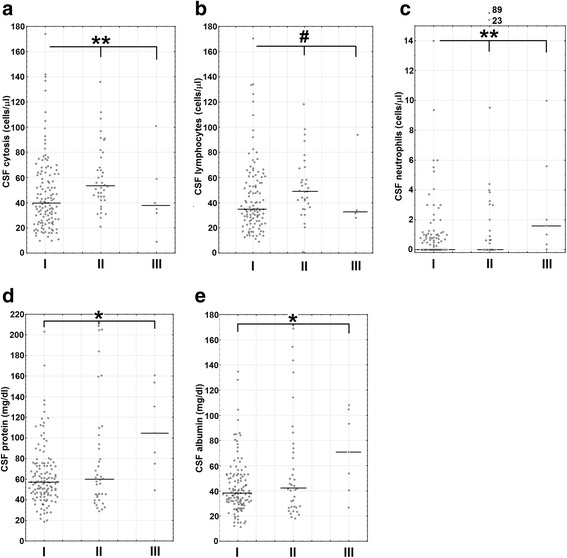
The convalescent cerebrospinal fluid parameters stratified by clinical outcome. The comparison of the cerebrospinal fluid (CSF) parameters in the control examination 10–16 days after the hospital admission between the groups of patients with full recovery (I, *n* = 121), patients with subjective complaints on follow-up (II, *n* = 38), and patients with long-lasting neurologic deficits (III, *n* = 7). The data from individual patients are shown with circles, median values with horizontal lines. Some individual data points in some plots may be lacking because of incomplete data. *A statistically significant trend with non-parametric ANOVA, *p* < 0.05. **The same with *p* < 0.01. ^#^A trend with a statistical significance at *p* = 0.056. **a** CSF total pleocytosis (cells/μl). **b** CSF lymphocyte count (cells/μl). **c** CSF neutrophil count (cells/μl). **d** CSF protein concentration (mg/dl). **e** CSF albumin concentration (mg/dl)

There were only 7 patients with objective neurologic sequelae in whom follow-up CSF differential cell count was available. Six of them had detectable neutrophils in convalescent CSF (constituting from 1 to 26% of the pleocytosis), and this association of the residual neutrophil infiltrate with persistent neurologic deficits was significant (*p* < 0.05) (Table 6). The median follow-up neutrophil count was significantly higher in neurologic sequelae group than in patients who fully recovered or had only persistent subjective complaints (*p* < 0.01), resembling trend observed for CSF albumin (*p* < 0.05) and total protein (*p* < 0.05), but contrasting with the pattern for total and lymphocytic pleocytosis (Fig. 3a–e). However, the follow-up neutrophil count did not correlate with simultaneous albumin and protein concentrations (a non-significant trend for a positive correlation could be observed in a subgroup of patients with neurologic sequelae only––not shown).

**Table 6:** The presence of the residual neutrophils in the cerebrospinal fluid of 172 TBE patients in the convalescent period after 10–16 days of hospital treatment is more frequent in patients with the objective neurologic sequelae

	TBE total *n* = 172	TBE sequelae	
		Absent *n* = 165	Present *n* = 7
Neutrophils absent (patient number and percentage)	99 58%	98^a^ 59%	1^a^ 14%
Neutrophils present (patient number and percentage)	73 42%	67^a^ 41%	6^a^ 86%

^a^Significant difference between the patients with and without sequelae (*p* < 0.05) in a chi-square test

### Prospective study

#### IL-8 concentration in the pilot study

**Fig. 4:**
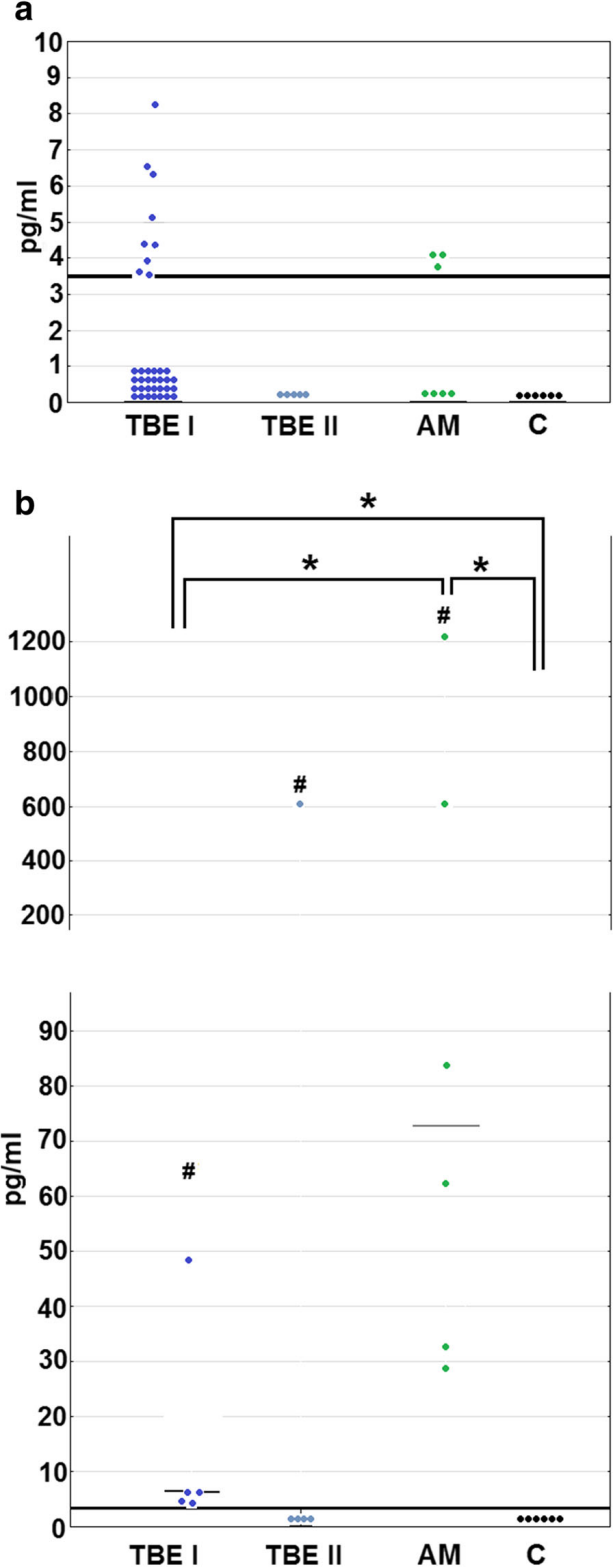
The concentration of IL-8 in serum and cerebrospinal fluid. **a** The concentration of IL-8 in serum from individual patients with tick-borne encephalitis (TBE; I—on admission to hospital, *n* = 36; II—on follow-up 10–14 days later, *n* = 5), other aseptic meningitis (AM; *n* = 7), and controls (C; *n* = 6). The thick horizontal line denotes the detection threshold. **b** The concentration IL-8 in cerebrospinal fluid (CSF) in the same groups as in **a**, except that only 5 TBE patients were included and the measurement from one AM patient was not available. The short horizontal lines denote the median values, when applicable, the thick horizontal line at the bottom—the detection threshold. Significant differences between the groups are shown by the lines above the plot with *p < 0.05. ^#^Significantly higher concentration in CSF in comparison with a simultaneous concentration in serum (*p* < 0.05). Note the difference of the scale between **a**, the lower, and the upper part of **b**

IL-8 was detected in 8 (22%) of TBE sera on admission and in none of the convalescent or control serum samples. It was detectable in all admission TBE CSF studied, in one convalescent CSF sample and in none of control CSF. IL-8 median concentration in admission CSF was significantly increased (*p* < 0.05) and manyfold higher than in serum (*p* < 0.05) (Fig. 4).

IL-8 was also detected in 3 (43%) of non-TBE meningitis sera and in all CSF samples. Its concentration in non-TBE meningitis CSF was significantly higher than in controls (*p* < 0.05), in TBE patients (*p* < 0.05), and in serum samples (*p* < 0.05) (Fig. 4).

#### IL-17, IL-22, and chemokine concentrations in the main study

**Fig. 5:**
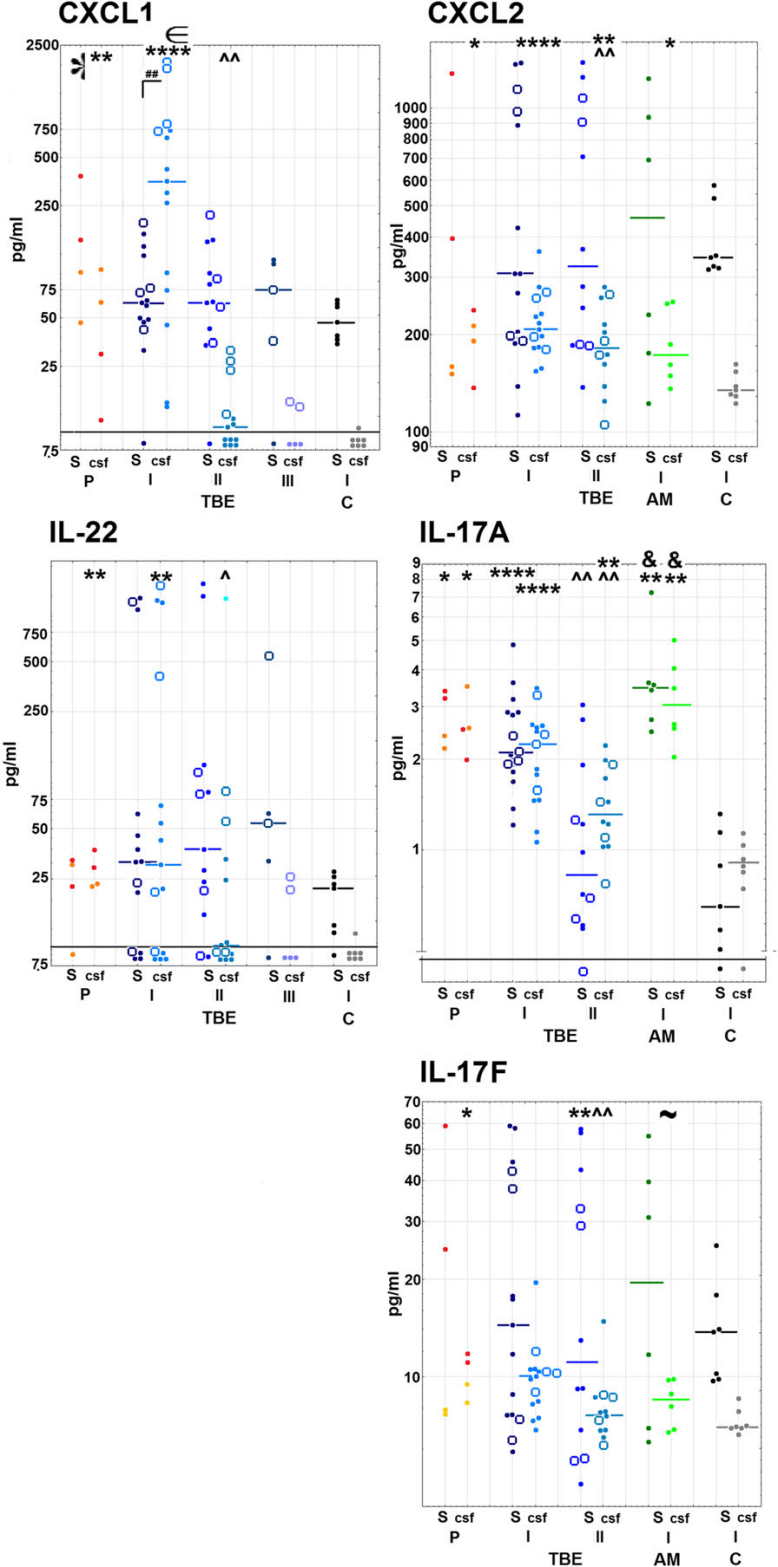
The concentrations of CXCL1, CXCL2, and Th17 cytokines in serum and cerebrospinal fluid. The concentrations of CXCL1, CXCL2, IL-17A, IL-17F, and IL-22 in serum (S) and cerebrospinal fluid (CSF) of patients in the neurologic phase of tick-borne encephalitis (TBE; *n* = 15), patients with non-TBE aseptic meningitis (AM; *n* = 6, CXCL2, IL-17A, and IL-17F only), and controls (C; *n* = 7). For TBE, the concentrations prior to the neurologic phase (P; *n* = 4, red symbols for patients 1 and 2 studied in the peripheral phase and orange symbols for patients 3 and 4 studied at the onset of the neurologic phase), in the active phase of the infection on admission to hospital (I), during early follow-up examinations before the discharge 10–14 days later (II; *n* = 13) and during late follow-up 4–8 weeks later (III; *n* = 5, CXCL1 and IL-22 only) are shown. Concentrations are expressed in pg/ml and presented in logarithmic scale. Individual data points are shown with dots and median values with short horizontal lines. In TBE group, 4 patients with clinically diagnosed encephalitis (as opposed to meningitis) are highlighted with empty symbols instead of filled dots. The thick horizontal lines at the bottom of panels denote the detection thresholds. Significant differences are highlighted by symbols at the top of each plot. *Significantly higher in comparison with controls (*p* < 0.05). **The same with *p* < 0.01. ****The same with *p* < 0.0001. ^∈^Significantly higher in TBE patients with meningoencephalitis than with meningitis (*p* < 0.05). ^Significant decrease in examination II in comparison with examination I (*p* < 0.05). ^^The same with *p* < 0.01. ^##^Significantly higher concentration in CSF than simultaneously in serum (*p* < 0.01). ^&^Significantly higher concentration in AM than simultaneously in TBE (*p* < 0.05). ^~^Higher concentration in TBE than simultaneously in AM with *p* = 0.066; half-star symbol in CXCL1 panel—concentration in TBE early serum samples higher than in C with *p* = 0.073

All the cytokines evaluated in the main study group were detectable in all or in a majority of the examination I TBE CSF and serum samples. The serum and CSF concentrations in individual patients tended to correlate (Additional file 2). Concentrations of CXCL1 and CXCL2 were significantly increased in admission CSF (*p* < 0.0001 for both) but not in serum. The CXCL1 median concentration in CSF was 5-fold and significantly higher than in serum (*p* < 0.01) while CXCL2 did not create a consistent concentration gradient towards CSF. The CXCL2 CSF/serum quotient was variable, in individual patients ranging from 0.16 to 1.62 (above 1.0 in four). The CXCL1 and CXCL2 CSF concentrations decreased in examination II (*p* < 0.01 for both), but CXCL2 remained elevated (*p* < 0.05) compared to controls. In patients examined during later follow-up (examination III), CXCL1 was undetectable in 3 out of 5 (Fig. 5, upper panels).

There was a significant increase of median concentrations of IL-17A (*p* < 0.0001), IL-17F (*p* < 0.01), and IL-22 (*p* < 0.01) in CSF of TBE patients, and IL-17A was significantly upregulated in serum as well (*p* < 0.0001). Their concentrations did not differ significantly between serum and CSF. All the concentrations decreased in examination II (*p* < 0.01), but IL-17A in CSF remained elevated in comparison with controls (*p* < 0.01). IL-22 in examination III was still detectable in 4 of 5 sera and (at low concentration) in 2 out of 5 CSF samples (Fig. 5, middle and lower panels).

On admission, CXCL1 concentration in CSF, but not in serum, was significantly higher in ME than in M patients (*p* < 0.01) and was the highest in a patient with the most severe clinical symptoms and altered consciousness. There was an analogous non-significant trend for IL-22, but not for IL-17A, IL-17F, and CXCL2 (Fig. 5).

CXCL2 CSF concentration in non-TBE meningitis was elevated (*p* < 0.05) and not different from the TBE group. Concentration of IL-17A was higher in serum and CSF of non-TBE patients than in controls (*p* < 0.01) and TBE (*p* < 0.05). IL-17F was not upregulated significantly in non-TBE meningitis, and its concentration in admission CSF tended to be lower than in TBE (*p* = 0.066) (Fig. 5, right side panels).

The cytokine concentrations measured in 4 patients in the peripheral phase or at the onset of the neurologic phase of TBE were comparable to the examination I values (Fig. 5, left side of each panel). The median concentration of IL-17A was significantly increased in serum (*p* < 0.05) and CSF (*p* < 0.05), and all the other cytokines were significantly upregulated in CSF. There was a tendency for higher Th17 cytokine concentrations than during examination I, with a trend being most notable for IL-17A and IL-17F in both serum and CSF (all *p* = 0.068). Two patients examined in the peripheral phase tended to have higher serum concentrations of the studied cytokines than the 2 examined later.

#### Intrathecal synthesis of the cytokines

**Fig. 6:**
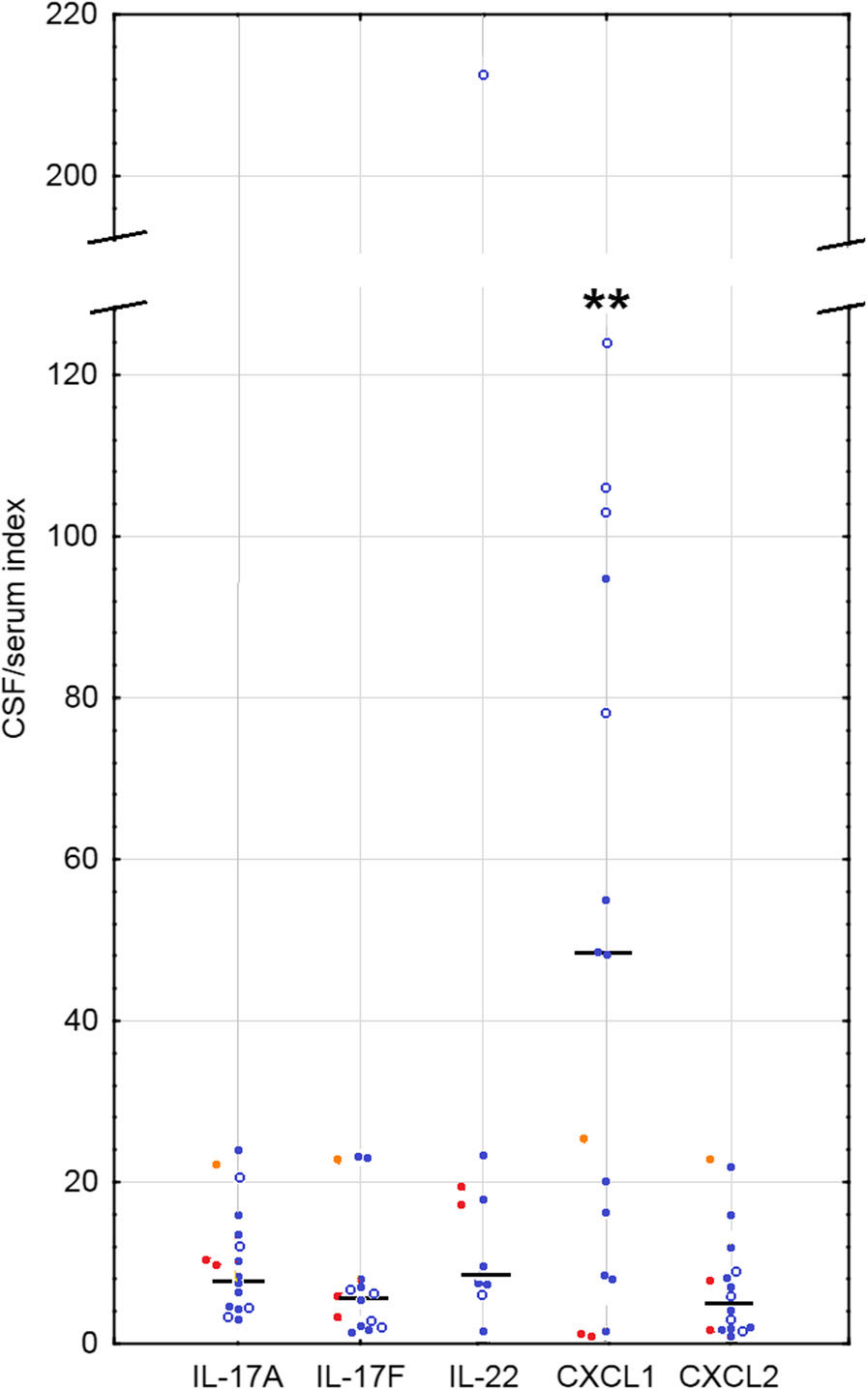
The cerebrospinal fluid concentration indexes of CXCL1, CXCL2, and Th17 cytokines The indexes of the cerebrospinal fluid (CSF) concentrations of chemokines CXCL1 and CXCL2 and interleukins IL-17A, IL-17F, and IL-22 in 14 TBE patients in the neurologic phase of the infection, calculated as described in the “Methods” section. Shown are values in individual patients (blue symbols) and median (black horizontal line), patients with meningoencephalitis are highlighted with circles and patients with meningitis with dots. The additional values obtained in 3 patients before the peak neurologic phase of TBE are shown to the left of each cytokine plot by colored points: red—patients 1 and 2 examined in the peripheral phase and orange—patient 4 examined early in the neurologic phase, before the maximum pleocytosis. Individual data points for some cytokines are missing because of the undetectable serum and/or CSF cytokine levels. **The significant difference between the meningitis and meningoencephalitis patients (*p* < 0.01).

Four of 5 TBE patients with a CSF IL-8 concentration measured had an undetectable IL-8 in serum and in the remaining patient *I*_IL-8_ was as high as 592, confirming intrathecal synthesis. All five cytokines examined in the main study group had median concentration indexes well above 1, with the median and most of the individual measurements highest for CXCL1, followed by IL-22 (Fig. 6).

*I*_CXCL1_ was significantly (*p* < 0.01) higher in ME than in M, with almost no overlap between the groups (Fig. 6).

CSF/serum indexes in the early pairs of samples could be assessed in patients 1, 2, and 4 (Fig. 6, points to the left of the main plots) and were generally in the range of examination I values and above the examination I median for IL-17A and IL-22. In patient 4, who had already developed detectable CSF pleocytosis, all the index values were either 2–3-fold higher than during examination I or non-calculable because of an undetectable serum concentration.

#### Correlations between parameters

**Fig. 7:**
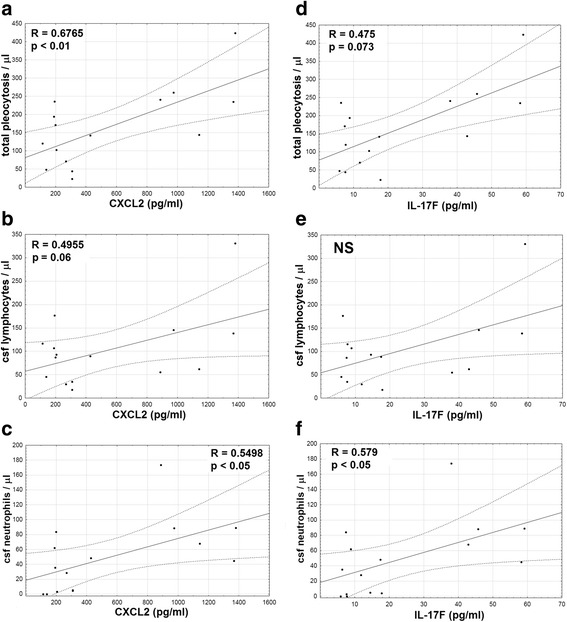
The correlations of cytokine concentrations in serum with pleocytosis. The correlation of the concentrations of CXCL2 (**a**–**c**) and IL-17F (**d**–**f**) in serum (expressed in pg/ml) of TBE patients on admission to hospital, presented on horizontal axes, with the cerebrospinal fluid total pleocytosis (**a**, **d**), lymphocyte count (**b**, **e**), and neutrophil count (**c**, **f**) (in cells/μl) on vertical axes. The data from the individual patients are shown with points, the linear fit with a continuous line and the 95% confidence interval with dashed lines. The strength *R* and statistical significance *p* of the correlations are shown directly on plots. NS non-significant

To address their pathogenetic role, we have assessed the correlations between the cytokine levels and the blood and CSF inflammatory parameters. The only significant correlation with the blood parameters was of the admission serum IL-17A concentration with CRP concentration (*p* < 0.05, *r* = 0.37; not shown). Additionally, serum IL-17F and CXCL2 correlated significantly with CSF neutrophil count (*p* < 0.05) and CXCL2 with total pleocytosis (*p* < 0.01) (Fig. 7).

**Fig. 8:**
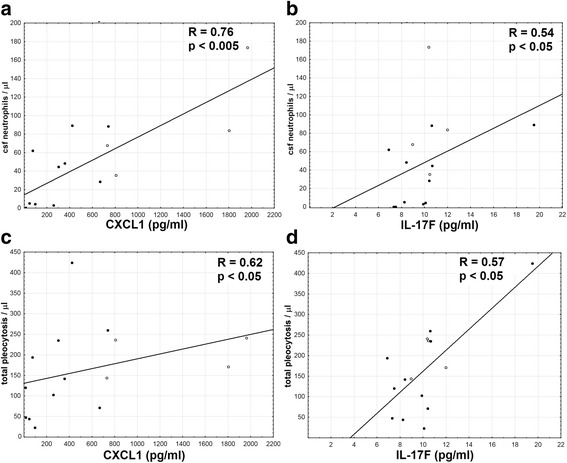
The correlation of the CXCL1 and IL-17F concentration in the cerebrospinal fluid with pleocytosis. The concentrations of CXCL1 (**a**, **c**) and IL-17F (**b**, **d**) in cerebrospinal fluid (CSF) of TBE patients obtained on admission to hospital are shown on horizontal axes, expressed in pg/ml, plotted against the cellular parameters of the admission CSF (in cells/μl) on vertical axes: neutrophil count in **a** and **b** and total pleocytosis in **c** and **d**. Data from the individual patients are shown with points (patients with meningitis) and circles (meningoencephalitis) and the best linear fit with a continuous line. The strength R and statistical significance p of the correlations are shown directly on plots

The CSF cytokine concentrations tended to correlate positively with total pleocytosis and CSF neutrophil count, which was significant for CXCL1 (*p* < 0.005 for a correlation with a neutrophil count) and IL-17F (*p* < 0.05) (Fig. 8, Additional file 3). There was no correlation with CSF lymphocyte count, total protein, and albumin concentration (Additional file 4). Analyzing the cytokine CSF/serum concentration quotients, as well as their CSF concentration indexes instead of the absolute concentrations did not change these results (not shown).

**Fig. 9:**
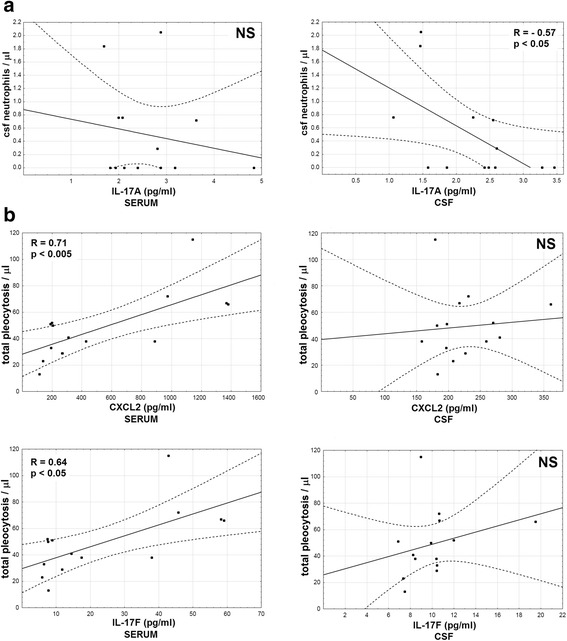
The correlations of the cytokine concentrations on admission with the convalescent cerebrospinal fluid parameters. The correlations between the cytokine concentrations on admission (in pg/ml, horizontal axes) and the cerebrospinal fluid (CSF) cellular parameters in follow-up examination 10–16 days after hospital admission (in cells/μl, on vertical axes). The data from the individual patients are presented with points, the best linear fit with a continuous line, and the 95% confidence interval with dashed lines. The strength *R* and statistical significance *p* of the correlations are shown directly on plots. NS non-significant. **a** Neutrophil counts plotted against IL-17A concentration in serum (left) and CSF (right). **b** Total pleocytosis plotted against CXCL2 (upper row) and IL-17F (lower row) in serum (left) and CSF (right)

There was no correlation of examination II cytokine levels with the simultaneous CSF parameters (not shown). Interestingly, there was a tendency for a negative correlation of the initial cytokine concentrations with the residual follow-up neutrophil counts, significant for IL-17A in CSF (*p* < 0.05, Fig. 9). On the other hand, serum IL-17F and CXCL2 concentrations on admission correlated with higher total and lymphocytic follow-up pleocytosis (*p* < 0.005 for the correlation of the admission CXCL2 in serum with follow-up pleocytosis, *p* < 0.05 for other) (Fig. 9).

## Discussion

The results of the retrospective study are in accordance with previous data and also give additional insights into the mechanism and consequences of the neutrophil CSF infiltration. The CSF neutrophil fraction was similar to previously described by Mickiené et al. in TBE, and somewhat lower than that reported in WNV-infected patient groups, which was 41% in meningitis and 45% in encephalitis according to Tyler et al., and 50% decreasing to 37% over 3 days since onset according to Rawal et al. [4, 19, 48]. In agreement with the trend observed in the later study, the presence of neutrophils in CSF of TBE patients was transient, giving a way to a convalescent phase pleocytosis of almost entirely mononuclear cells. The CSF neutrophil count did not correlate with a C-reactive protein concentration, suggesting it was not directly dependent on the systemic inflammatory response, and although it could be facilitated by high numbers of circulating neutrophils, it was not evidently dependent on neutrophilia and probably driven by other, likely intrathecal, factors. The presence of neutrophils in CSF was not associated with the BBB disruption, and it did not correlate with CSF lymphocyte count, pointing to independent mechanisms behind the migration of these two cell populations into CSF. In the WNV encephalitis model described by Bai et al., neutrophils co-accumulated with T CD8+ lymphocytes and the migration of the later was partly dependent on CXCL1 and CXCL2, either because of their direct response to these chemokines or because of the cooperation and co-migration with neutrophils [27]. Although such a relation with a particular population of lymphocytes would be of pathogenetic significance, it could not be evaluated in our retrospective data and requires further studies. Taken together, our results suggest an early massive recruitment of neutrophils into CSF, not dependent directly on the peripheral inflammation, lymphocyte influx, and BBB function, and possibly facilitated by, but not critically dependent on the presence of an increased population of circulating neutrophils.

The data on the pathogenetic role of the neutrophilic CNS infiltration in *Flavivirus* encephalitis come mainly from animal models and remain conflicting. In the mouse model of viral encephalitis studied by Zhou et al., neutrophils constitute the first population of cells infiltrating CNS and their depletion leads to an increased virus replication and impaired migration of mononuclear cells. The authors hypothesized that neutrophils initiate the protective accumulation of mononuclear cells within CNS by first contributing to BBB disruption and then by secreting chemokines including CCL3, CCL4, CXCL9, CXCL10, and CXCL11 [25], some of which have actually been detected in TBE CSF [13, 49]. On the other hand, a number of studies suggest a role of neutrophils in the development of the severe *Flavivirus* encephalitis, which could be mediated either by an increased viral load entering CNS with migrating neutrophils or by their direct contribution to the intrathecal immunopathology. In mice infected with Murray Valley encephalitis virus, both the depletion of the neutrophils dominating in the brain infiltrate and the inhibition of the inducible nitric oxide synthase (iNOS) prolong the survival, suggesting an immunopathology dependent on the neutrophil-generated NO [26]. In the JE model, the severity of encephalitis is related to skewing of the specific cellular response from regulatory (Treg) to Th17-type, with the increased IL-17, CXCL1, and CXCL2 expression promoting the CNS infiltration by granulocytes, monocytes, and cytotoxic CD8+ lymphocytes [50]. In mice infected with Langat virus, a prolonged neutrophil influx into CNS is a hallmark of a severe encephalitic phenotype [51]. The Bai et al. study on WNV encephalitis mouse model suggests a more complicated scenario, with infected neutrophils contributing to the virus entry into CNS, but later playing a protective role during an established CNS infection [27]. However, as the survival of WNV infection in mice lacking IL-22 and CXCR2 signaling is improved, the overall effect of neutrophil infiltration seems unfavorable [9, 29, 52].

The significance of these observations for the pathogenesis of human *Flavivirus* encephalitis remains unclear. In WNV patients described by Tyler et al., the CSF parameters did not differentiate meningitis from encephalitis and although a higher pleocytosis, neutrophil fraction, and CSF protein concentration correlated with a worse outcome, the prognostic value of individual factors was weak [19]. In the study of Mickiené et al., there was no significant association of CSF neutrophils with the severity of TBE, although the data presented by the authors may suggest a non-significant trend towards higher neutrophil fraction in more severely ill patients [4]. The sensitivity of our own analysis was limited by its retrospective character and by a small number of patients with severe meningoencephalitis. The peripheral neutrophilia tended to be the highest in patients with the most severe neurologic presentation and with permanent neurologic sequelae, consistent with a possible role of circulating neutrophils in a virus spread and migration into CNS in accordance with animal models [10, 27], but a small number of patients and possible confounding factors in severely ill subjects make this observation inconclusive. The CSF neutrophils on admission did not correlate with clinical presentation, severity, or outcome of the CNS involvement, with an exception of a higher neutrophil count in patients with myelitis, hinting at the specific pathogenesis of the spinal involvement in TBE. The presence of the residual neutrophils in the convalescent CSF was associated with encephalitis and with worse neurologic outcome, which may reflect a prolonged intrathecal inflammation in more severe TBE cases, but it is not clear if neutrophils played any specific pathogenetic role at this late stage of the disease. Interestingly, it resembles the results of a Langat virus study by Michlmayr et al. in which neutrophil counts within CNS were initially similar between resistant and sensitive mice, but differed at the later stage of the encephalitis [51].

To identify mediators of the neutrophilic response in TBE, we have first verified the synthesis of IL-8 described in previous studies. IL-8 was previously reported in CSF of patients with aseptic meningitis of various etiology [53, 54], and its upregulation has been well documented in JEV infection: it has been detected in serum of persons receiving live attenuated anti-JEV vaccine [55] and in serum and CSF of JE patients, in whom it correlates both with the neutrophil counts and with the disease severity [29, 56, 57]. Recently, Palus et al. have confirmed increased IL-8 concentration in serum of TBE patients [47] and Pietikäinen described its moderate increase in their CSF [46], although no direct comparison of serum and CSF levels was made. Our study confirmed that IL-8 is upregulated mainly intrathecally, creating a steep concentration gradient between CSF and serum, consistent with its role as a chemotactic factor.

Following the IL-8 detection, we have studied expression of two additional chemokines for neutrophils (CXCL1 and CXCL2), as well as of Th17 cytokines previously detected in animal models of *Flavivirus* encephalitis [9, 27, 50, 58, 59]. In general, our findings confirm the intrathecal synthesis of all these mediators and suggest their contribution to CNS neutrophilic infiltration. The concentrations of CXCL1 in CSF, of IL-17F in both serum and CSF, and of CXCL2 in serum correlated specifically with CSF neutrophil count, but not with the lymphocyte count or markers of BBB integrity. As particular cytokines presented with different dynamics of expression and concentration gradients between serum and CSF, their role in the pathogenesis might vary. Increased concentration of IL-17A in serum suggests its involvement in the peripheral response, while IL-22 expression was mainly intrathecal. Of the CXCR2-agonistic chemokines, CXCL1, but not CXCL2, created a chemotactic gradient towards CSF and correlated significantly with the CSF neutrophil count.

Besides IL-8, there are few data on the expression of Th17 cytokines and chemokines for neutrophils in other human CNS infections to provide context for our TBE observations. According to Pietikäinen et al., the concentration of IL-17 is higher in CSF of TBE patients in comparison with neuroborreliosis, a condition characterized by almost exclusively lymphocytic pleocytosis [46]. Zhang et al. described increased IL-22 and decreased IL-17A levels in serum of children with enterovirus encephalitis qualitatively different from the pattern of response we detect in TBE [60]. In our study, of cytokines compared between TBE and non-TBE meningitis groups, the CSF concentration of IL-8 and IL-17A was lower in TBE, of IL-17F tended to be higher, and of CXCL2 did not differ. Thus, although non-TBE patients presented with a clearly different clinical picture of mild, quickly resolving, and uncomplicated meningitis, with no peripheral neutrophilia and mononuclear CSF infiltrate, the observed profile of the expression of Th17-related cytokines do not explain these differences. The differentiating factors, however, could be pro-neutrophilic cytokines not investigated in our study, like CXCL5, or not included in this part of the analysis: IL-22 and CXCL1.

Both our data and previous animal results point to an important role of IL-22 in the pathogenesis of *Flavivirus* neuroinfection, particularly in mediating the neutrophil migration. In the mouse model of WNV encephalitis, the lack of IL-22 expression decreased CNS viral load, infiltration by neutrophils, and mortality rate, attenuating all the aspects of the intrathecal infection and inflammation. However, when WNV was injected intrathecally, IL-22 was moderately protective [9]. These results show that IL-22 signaling promotes the onset of *Flavivirus* encephalitis but later contributes to the intrathecal protective response, which resembles the sequential role of neutrophils in WNV infection as described by Bai et al. [9, 27]. These effects of IL-22 are mediated by an upregulation of CXCR2 on neutrophils and the intrathecal expression of CXCL1 and CXCL5 [9]. According to Michlmayr et al., an increase of mRNA levels for CXCL1 and CXCL2 in the brains of WNV-infected mice may be as high as 1000-fold [58]. In vitro, human brain microvasculature endothelial cells secrete CXCL1 and CXCL5 and favor transmigration of neutrophils under IL-22 stimulation [9], confirming that findings from animal experiments may apply to human encephalitis. In TBE, we have observed an intrathecal expression of IL-22 and especially of CXCL1, the later associating significantly both with the encephalitic presentation and with high CSF neutrophil counts and thus reproducing important features of these animal and in vitro models.

These results may have implications for a better understanding of a still debatable route of TBEV entry into CNS, a crucial event differentiating between the mild peripheral and potentially severe neuroinvasive infection. In vitro TBEV is able to cross BBB by infecting and replicating persistently within brain endothelial cells, without directly affecting BBB structure and permeability [6]. This forms a likely route of the initial CNS entry during the peripheral phase, but additional migration mechanisms that could increase CNS viral load are possible and supported by mouse WNV encephalitis models [7–9]. Previously, we have studied the postulated mode of CNS entry following BBB disruption by a systemic inflammatory response mediated by macrophage migration inflammatory factor (MIF) and tumor necrosis factor alpha (TNFα)––a mechanism that seems feasible but remains unproven and does not exclude the existence of other pathogenically and clinically relevant routes of infection [61]. The current results are consistent with the alternative possibility of the migration of the infected neutrophils dependent on intrathecal IL-22 and CXCL1 as a clinically important route of TBEV entry into CNS. This transition to the neurologic phase of TBE must be determined during a peripheral phase and a study of the cytokine expression in patients at that stage of the disease, as well as in patients with a flu-like TBEV infection not progressing to neurologic phase, could be highly informative as for factors determining the neuroinvasion. However, such study is very difficult to arrange in a clinical setting, as a TBEV etiology is only rarely suspected at that stage of the disease. Our data from 4 patients seen in the peripheral or at the onset of the neurologic phase, although limited, offer a unique insight into this key period of the TBEV infection. The peripheral phase cytokine and chemokine concentrations were at least comparable with neurologic phase values and for Th17 cytokines tended to be even higher. The early systemic Th17-type response could facilitate TBEV neuroinvasion in both postulated mechanisms, contributing to BBB disruption caused by the systemic inflammation while in the same time providing a pool of circulating neutrophils able to infiltrate CNS. In animal experiments, CXCR2 signaling contributes to the neutrophil migration from the bone marrow into blood under stress stimuli [62] and IL-22 causes neutrophilia mediated by CXCL1 release from hepatocytes [37, 43], and similar mechanisms could play a role in neutrophil mobilization and migration in TBE patients, contributing to a viral spread within infected cells. More surprisingly, the cytokines tended to be already upregulated intrathecally in 2 TBE patients with peripheral infection, before the onset of any changes in the general CSF examination, suggesting that the intrathecal inflammatory process undetectable with standard diagnostic techniques had already been initiated. Possibly, Th17 cytokines in CSF could be even applied as prognostic markers of a developing neuroinfection in patients with a suspected TBE and an unremarkable result of the general CSF examination.

We found the concentrations of pro-neutrophilic cytokines to decrease in the early convalescent period of TBE, confirming the association of a Th17 response with its early stages. However, this early response had a lasting influence possible to trace in the convalescent CSF parameters. Especially, the CSF concentration of IL-17A correlated negatively with the residual CSF neutrophil infiltrate, which in the retrospective study associated with a more severe presentation. According to Zhou et al. the protective mononuclear CNS infiltrate is facilitated by neutrophils, but once it is established, it may suppress further neutrophil migration, for example, through effects of interferon gamma (IFN-γ) on the local chemokine synthesis [25]. According to this model, the initial vivid Th17-type inflammation should be short-lived and associated with a good recovery, which is consistent with a coexistence of an initial high IL-17 expression, prompt elimination of neutrophils from CSF, and a good clinical outcome we observe. This may document a secondary protective effect of the neutrophilic response in encephalitis analogous to that described by Bai et al. in the animal model [27]. Thus, the complex roles played by neutrophils in animal models seem to be observable in a human TBE as well. This may explain the lack of a straightforward correlation of the neutrophilic response with the clinical severity, as different protective and pathogenic effects may be difficult to trace and disentangle in individual patients.

## Conclusions

Neutrophils migrate into CSF of patients with TBE independently of the lymphocyte influx and BBB disruption. The CSF neutrophil count does not differ between meningitis and meningoencephalitis, but is higher in myelitis, and normalizes with delay in patients with encephalitis and with persistent neurologic complications, suggesting a complex involvement in the intrathecal pathology. The pro-neutrophilic Th17 cytokines and chemokines signaling through CXCR1 and CXCR2 are upregulated intrathecally, and at least some of them (IL-17A) also systemically, starting from the peripheral and continuing into the neurologic phase of TBE, which allows them to exert their influence during the critical events deciding about the onset and severity of the CNS involvement. Of Th17 chemokines, IL-22 is most vividly expressed intrathecally. Of chemokines attracting neutrophils at least IL-8 and CXCL1 create chemotactic gradients towards CSF and may be responsible for neutrophil recruitment, and CXCL1 concentration correlates with CSF neutrophil count and with encephalitic presentation. By analogy with animal models, a signaling axis involving IL-22, its mediator chemokines and CXCR1/CXCR2 receptors may increase the TBEV influx into CNS with infected neutrophils, thus providing an important route of neuroinvasion leading to a more severe CNS involvement. After the encephalitis is established, however, an intrathecal neutrophilic response may be protective, contributing to its control and eradication.

## Additional files

Additional file 1: Correlation between the time since onset of fever and peripheral blood neutrophil count. The time since the onset of fever till the admission to hospital as reported by a patient and recorded in medical documentation (in days) is presented on horizontal axis and the neutrophil count on admission (in cells/μl) on vertical axis. The data from the individual patients are shown with crosses, the linear fit with a continuous line and the 95% confidence interval with dashed lines. The strength *R* and significance *p* of the correlation is given in the upper right corner. (BMP 2655 kb)

Additional file 2: Correlations between the serum and cerebrospinal fluid concentrations CXCL1, CXCl2, and Th17 cytokines. Concentrations of IL-17A, IL-17F, IL-22, CXCL1, and CXCL2 in serum (S) samples obtained from tick-borne encephalitis patients on admission to hospital are shown horizontal axes and in cerebrospinal fluid (CSF) samples obtained simultaneously on vertical axes (expressed in pg/ml). The data from individual patients are shown with points (patients with meningitis) and circles (meningoencephalitis), the linear fit with a continuous line, and the 95% confidence interval with dashed lines. The strength *R* and statistical significance *p* of the linear fit are shown in the upper right corner of each panel. NS not significant. (BMP 19542 kb)

Additional file 3: The lack of a significant correlation between the cerebrospinal fluid concentrations of IL-17A, IL-22 and CXCL2 and the cerebrospinal fluid pleocytosis and neutrophil count. The plots presenting cerebrospinal fluid (CSF) total pleocytosis (left) and neutrophil count (right) in tick-borne encephalitis patients on admission to hospital on vertical axes (expressed in cells/μl), plotted against CSF concentrations on cytokines: IL-17A (upper row), IL-22 (middle row), and CXCL2 (lower row) presented on horizontal axes (expressed in pg/ml). The data from individual patients are shown with points (patients with meningitis) and circles (meningoencephalitis), the linear fit with a continuous line. The apparent trend for a positive correlation for IL-17A and CXCL2 did not reach the level of the statistical significance. NS non-significant. (BMP 20257 kb)

Additional file 4: The lack of a significant correlation of the cerebrospinal fluid concentrations of the studied cytokines with the cerebrospinal fluid lymphocyte count, total protein, and albumin concentration. The results for IL-17F and CXCL1 are shown, representative for all the cytokines studied. The cerebrospinal fluid parameters in tick-borne encephalitis patients on admission to hospital are shown on vertical axes: lymphocyte count expressed in cells/μl (left), total protein concentration expressed in mg/dl (center), albumin concentration in mg/dl (right), plotted against the simultaneous CSF cytokine concentrations presented on horizontal axes, expressed in pg/ml: IL-17F in the upper and CXCL1 in the lower row. The data from individual patients are shown with points (patients with meningitis) and circles (meningoencephalitis), the linear fit with a continuous line. The trend for a positive correlation apparent on some plots did not reach the level of the statistical significance. NS non-significant. (BMP 20761 kb)

## Abbreviations

AM: Aseptic meningitis
BBB: Blood-brain barrier
CNS: Central nervous system
CRP: C-reactive protein
CSF: Cerebrospinal fluid
G-CSF: Granulocyte colony-stimulating factor
IFN: Interferon
IL: Interleukin
JE: Japanese encephalitis
JEV: Japanese encephalitis virus
M: Meningitis
ME: Meningoencephalitis
MEM: Meningoencephalomyelitis
MIF: Macrophage migration inhibitory factor
TBE: Tick-borne encephalitis
TBEV: Tick-borne encephalitis virus
TNFα: Tumor necrosis factor alpha
WNV: West Nile virus
