# Supplementary figures and images for: The intrathecal expression and pathogenetic role of Th17 cytokines and CXCR2-binding chemokines in tick-borne encephalitis
Source: J Neuroinflammation. 2018 Apr 20;15:115. doi: 10.1186/s12974-018-1138-0 (PMC5909263; doi:10.1186/s12974-018-1138-0)

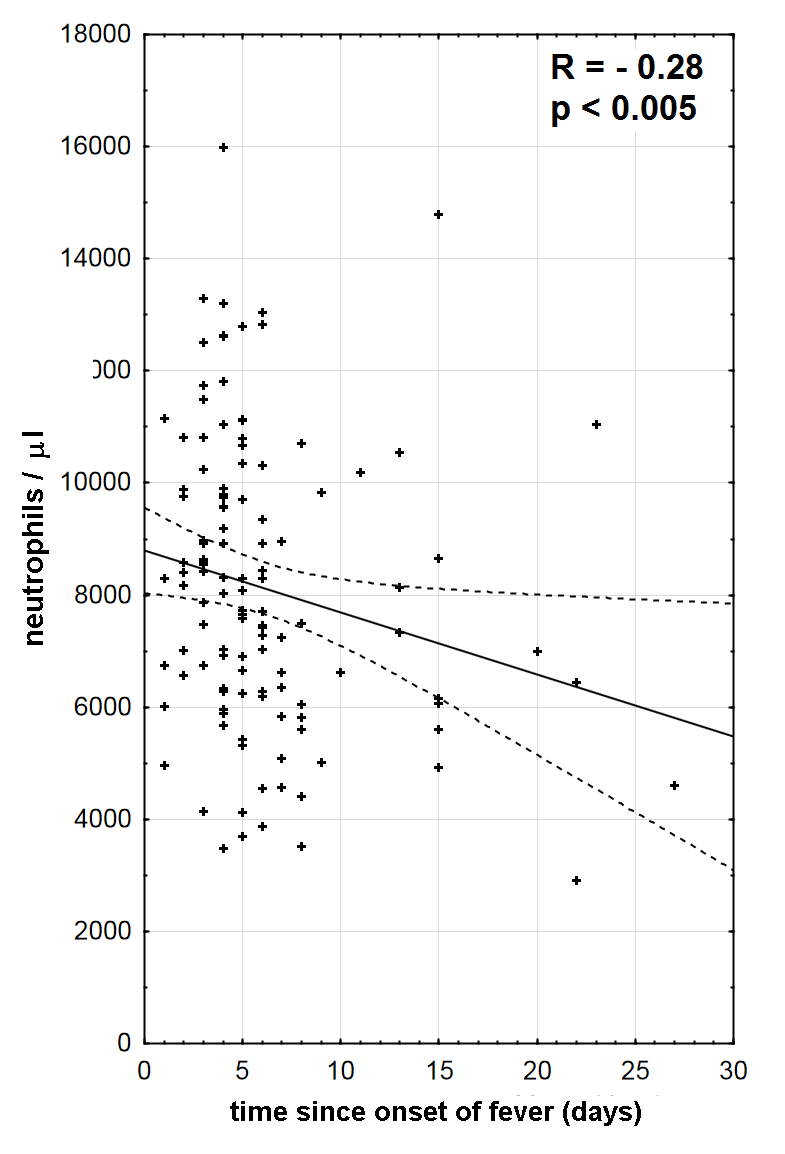

Supplement: Supplementary file 1 — Correlation between the time since onset of fever and peripheral blood neutrophil count. The time since the onset of fever till the admission to hospital as reported by a patient and recorded in medical documentation (in days) is presented on horizontal axis and the neutrophil count on admission (in cells/μl) on vertical axis. The data from the individual patients are shown with crosses, the linear fit with a continuous line and the 95% confidence interval with dashed lines. The strength R and significance p of the correlation is given in the upper right corner. (BMP 2655 kb) [file 12974_2018_1138_MOESM1_ESM.bmp]

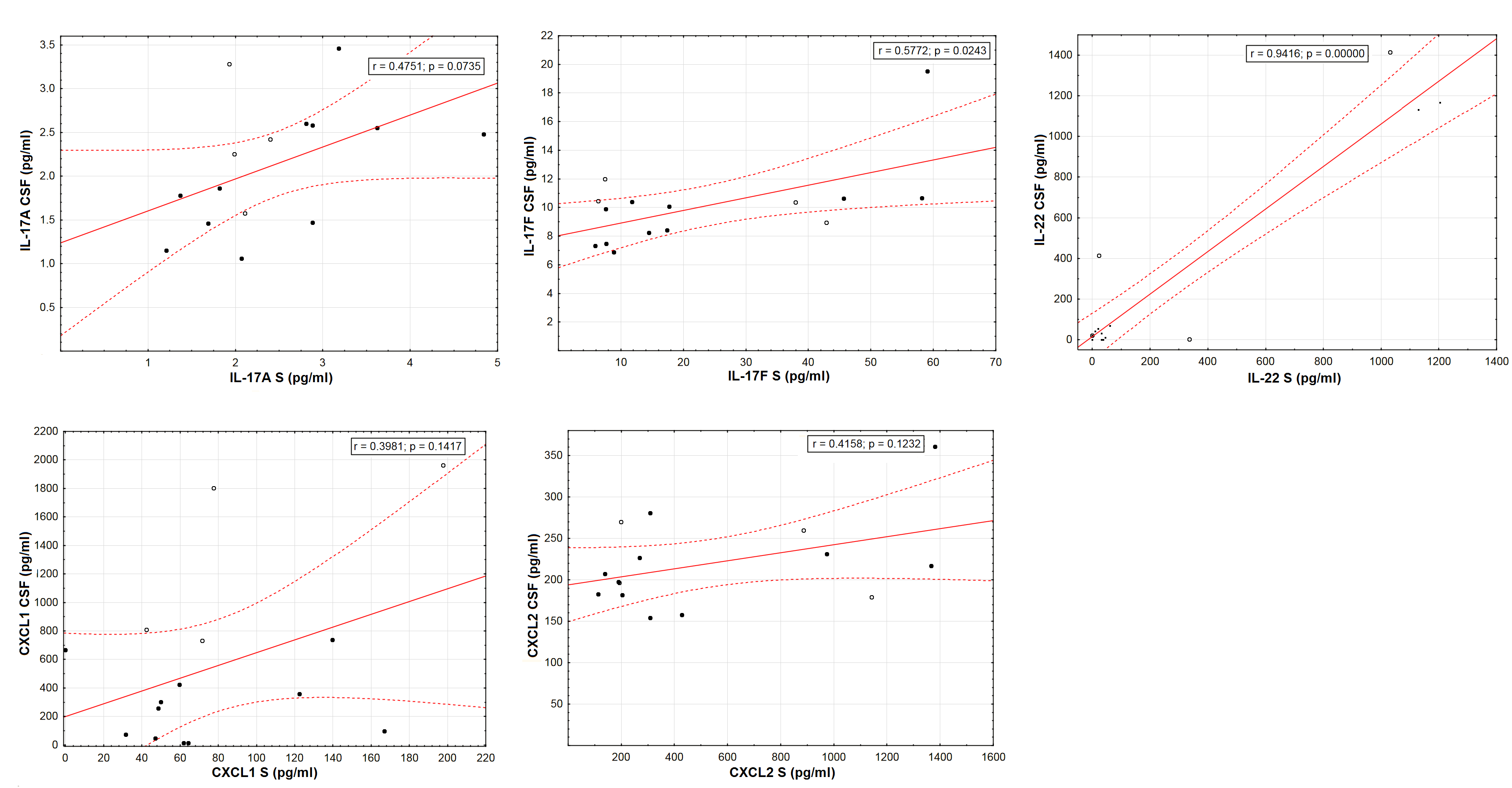

Supplement: Supplementary file 2 — Correlations between the serum and cerebrospinal fluid concentrations CXCL1, CXCl2, and Th17 cytokines. Concentrations of IL-17A, IL-17F, IL-22, CXCL1, and CXCL2 in serum (S) samples obtained from tick-borne encephalitis patients on admission to hospital are shown horizontal axes and in cerebrospinal fluid (CSF) samples obtained simultaneously on vertical axes (expressed in pg/ml). The data from individual patients are shown with points (patients with meningitis) and circles (meningoencephalitis), the linear fit with a continuous line, and the 95% confidence interval with dashed lines. The strength R and statistical significance p of the linear fit are shown in the upper right corner of each panel. NS not significant. (BMP 19542 kb) [file 12974_2018_1138_MOESM2_ESM.bmp]

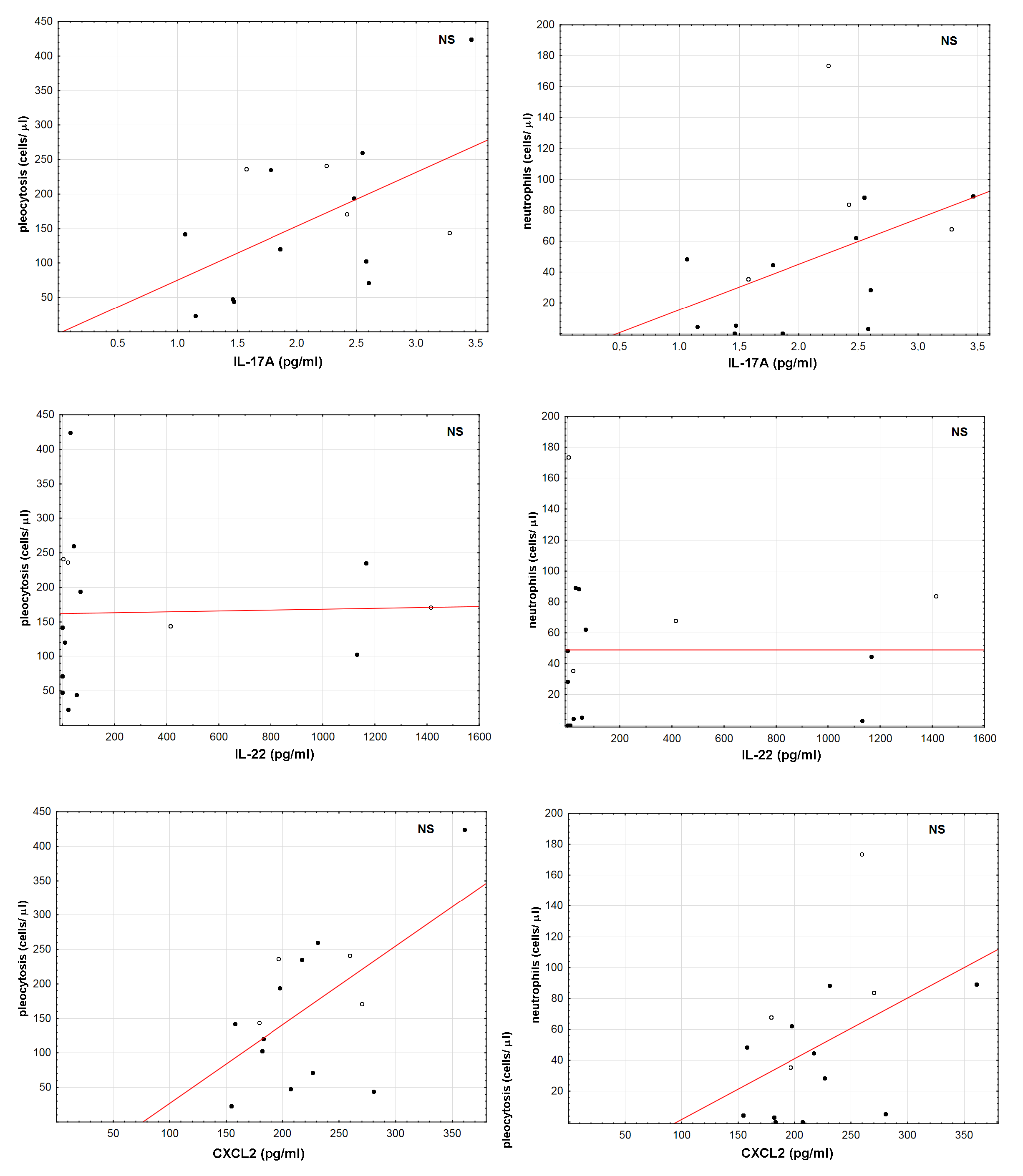

Supplement: Supplementary file 3 — The lack of a significant correlation between the cerebrospinal fluid concentrations of IL-17A, IL-22 and CXCL2 and the cerebrospinal fluid pleocytosis and neutrophil count. The plots presenting cerebrospinal fluid (CSF) total pleocytosis (left) and neutrophil count (right) in tick-borne encephalitis patients on admission to hospital on vertical axes (expressed in cells/μl), plotted against CSF concentrations on cytokines: IL-17A (upper row), IL-22 (middle row), and CXCL2 (lower row) presented on horizontal axes (expressed in pg/ml). The data from individual patients are shown with points (patients with meningitis) and circles (meningoencephalitis), the linear fit with a continuous line. The apparent trend for a positive correlation for IL-17A and CXCL2 did not reach the level of the statistical significance. NS non-significant. (BMP 20257 kb) [file 12974_2018_1138_MOESM3_ESM.bmp]

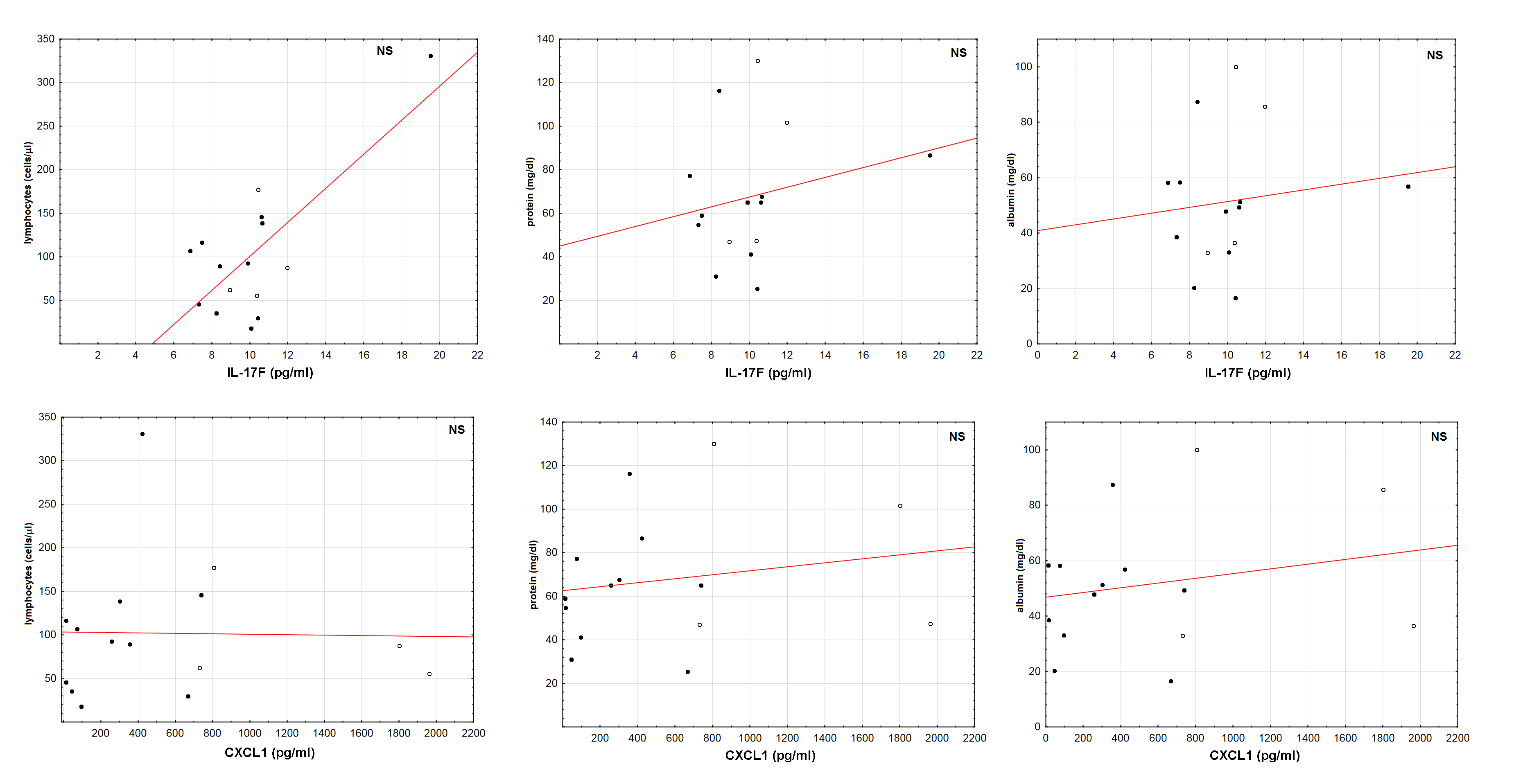

Supplement: Supplementary file 4 — The lack of a significant correlation of the cerebrospinal fluid concentrations of the studied cytokines with the cerebrospinal fluid lymphocyte count, total protein, and albumin concentration. The results for IL-17F and CXCL1 are shown, representative for all the cytokines studied. The cerebrospinal fluid parameters in tick-borne encephalitis patients on admission to hospital are shown on vertical axes: lymphocyte count expressed in cells/μl (left), total protein concentration expressed in mg/dl (center), albumin concentration in mg/dl (right), plotted against the simultaneous CSF cytokine concentrations presented on horizontal axes, expressed in pg/ml: IL-17F in the upper and CXCL1 in the lower row. The data from individual patients are shown with points (patients with meningitis) and circles (meningoencephalitis), the linear fit with a continuous line. The trend for a positive correlation apparent on some plots did not reach the level of the statistical significance. NS non-significant. (BMP 20761 kb) [file 12974_2018_1138_MOESM4_ESM.bmp]
